# Coronary computed tomography angiography using the diluted contrast material protocol: a technique for achieving uniform coronary artery enhancement

**DOI:** 10.1007/s11604-025-01845-y

**Published:** 2025-07-30

**Authors:** Kentaro Ohara, Kazuki Yoshida, Hikaru Nishiyama, Yuki Tanabe, Yusuke Kobayashi, Naoto Kawaguchi, Megumi Matsuda, Kaito Okamoto, Shiori Utsunomiya, Teruhito Kido

**Affiliations:** Department of Radiology, Ehime University Graduate School of Medicine, Toon, Ehime Japan

**Keywords:** Computed tomography, Contrast material, Coronary attenuation, Injection protocol, Coronary CT angiography

## Abstract

**Purpose:**

A diluted contrast material (CM) protocol is used to achieve consistent coronary artery enhancement. In this study, we aimed to evaluate the feasibility of the diluted CM protocol compared with the fractional dose (FD) protocol for coronary computed tomography angiography (CCTA) performed at a low tube voltage (100 kVp).

**Materials and Methods:**

We analyzed 103 patients (mean age, 68 ± 14 years; 54 males) who underwent CCTA between July 2022 and September 2024. A total of 50 and 53 patients underwent the diluted CM and the FD protocols, respectively. The diluted CM protocol involves individualized contrast dilution based on a simulated time-attenuation curve to attain a target arterial attenuation of 450 Hounsfield units (HU). In the FD protocol, contrast material volume and injection rate were determined using the patient’s body weight. The aortic and coronary attenuation values were measured and compared. Subsequently, the variability in enhancement and iodine dose per body weight was analyzed.

**Results:**

The mean coronary artery attenuation across all coronary segments was significantly higher in the diluted CM protocol group than the FD protocol group (424.0 ± 26.0 HU vs. 393.0 ± 59.4 HU, *P* < 0.001). The diluted CM protocol demonstrated significantly lower variability in contrast enhancement across all coronary segments.

**Conclusion:**

The diluted CM protocol resulted in more consistent coronary enhancements compared with the FD protocol during CCTA performed at a tube voltage of 100 kVp. This suggests its potential value in optimizing image quality.

**Supplementary Information:**

The online version contains supplementary material available at 10.1007/s11604-025-01845-y.

## Introduction

Coronary computed tomography angiography (CCTA) has become a widely adopted first-line imaging modality for evaluating patients with chronic coronary diseases [1–3]. CCTA demonstrates high diagnostic accuracy for detecting substantial coronary stenosis (> 50%) when compared with invasive coronary angiography, with a sensitivity of 95–99%, specificity of 68–93%, positive predictive value of 75–93%, and negative predictive value of 96–99% [4].

The degree of coronary artery enhancement critically influences the assessment of coronary artery stenosis severity. The optimal range of coronary artery enhancement is 350–500 Hounsfield units (HU) [5]. Excessive coronary artery enhancement (≥ 500 HU) may lead to underestimation of coronary artery stenosis severity, whereas insufficient coronary artery enhancement (< 250 HU) can cause overestimation [5, 6]. Therefore, achieving and maintaining consistent coronary artery enhancement is essential in preserving the high diagnostic performance of CCTA.

Commonly used techniques for scan timing include bolus tracking (BT) and test bolus (TB) protocols [7]. Although the BT protocol offers simplicity, it is more vulnerable to variations in individual cardiovascular functions, which can result in suboptimal scan timing, particularly in patients with cardiac dysfunction [8]. Conversely, the TB protocol allows for more precise timing by evaluating individual contrast dynamics; however, it requires two contrast injections, making the procedure more complex [7]. In addition to scan timing, proper adjustment of the contrast material dosage is important. The fractionated dose (FD) protocol, which considers the patient's body size and injection rate, is widely used for this purpose.

Recently, the role of CCTA has expanded beyond coronary artery stenosis evaluation to include coronary artery plaque characterization and fat attenuation index (FAI) assessment, both of which are important prognostic indicators [9, 10]. Consequently, maintaining a stable and reproducible coronary artery enhancement has become increasingly important. Techniques such as test bolus tracking and diluted contrast material (CM) protocols can achieve more consistent coronary artery enhancement [11, 12]. However, previous studies assessing these methods were primarily conducted at a tube voltage of 120 kVp. Limited evidence remains regarding their effectiveness at the currently recommended low tube voltage of 100 kVp for patients with a body mass index (BMI) of < 30 [13].

In this study, we aimed to evaluate the efficacy of the diluted CM protocol compared with the FD protocol at a low tube voltage (100 kVp).

## Materials and methods

### Study design and population

A total of 130 consecutive patients with stable angina who underwent CCTA using a third-generation dual-source computed tomography (CT) scanner (SOMATOM Force; Siemens Healthineers, Erlangen, Germany) and an automatic dual injector (DUAL SHOT GX7; Nemoto Kyorindo, Tokyo, Japan) between July 2022 and September 2024 were retrospectively enrolled. The exclusion criteria were as follows: (1) arrhythmia, (2) inadequate breath-holding, (3) severe calcification in all coronary arteries, (4) subcutaneous extravasation of contrast material, and (5) contrast material injection protocols other than the diluted CM or FD protocols. This retrospective study was approved by the Institutional Ethics Committee of Ehime University Hospital (No. 2504006), which waived the requirement for informed consent.

### CCTA acquisition protocol

All patients received 0.6 mg of nitroglycerin spray administered via two puffs (Myocor; TOA EIYO, Tokyo, Japan) prior to scanning. In addition, when the resting heart rate (HR) exceeded 75 beats per min (bpm), 3–10 mg of β-blocker (Landiolol; ONO PHARMACEUTICAL, Osaka, Japan) was administered intravenously 5 min before the timing.

The timing bolus scan was initiated 6 s after commencing the CM injection and was continued until approximately 7–8 s after the time to peak enhancement. Axial data were acquired at 1 s intervals at the level of the ascending aorta with the following parameters: gantry rotation time, 0.25 s/rotation; tube voltage, 100 kVp; tube current, 20 mA; and collimation, 1 × 10 mm. The real scan parameters were as follows: retrospective electrocardiogram-gated scan mode, with dose-modulation targeting a phase of 45% of the RR interval (heart rate ≥ 75 bpm, or prospective-triggered scan mode, targeting a phase of 75% of the RR interval (heart rate < 75 bpm); tube voltage, 100 kVp; reference mAs, 216 mAs/rot; gantry rotation time, 0.25 s/rotation; pitch factor, 0.15; collimation, 2 × 192 × 0.6 mm; 200 mm display field of view; and 512 × 512 image matrix. Axial images were constructed with a slice thickness of 0.75 mm and a section interval of 0.4 mm using a medium soft convolution kernel (Siemens Bv40) and model-based iterative reconstruction (ADMIRE, Siemens Healthineers) at a strength level of two.

### Contrast material injection protocol

The diluted CM and FD protocols used either ioversol (Optiray 320 mg iodine/mL; Guerbet Japan, Tokyo, Japan) or iopamidol (Iopamiron 370 mg iodine/mL; Bayer Yakuhin, Osaka, Japan).

### Diluted CM protocol

Initially, a timing bolus scan was conducted to optimize scan timing and determine the appropriate contrast material dilution rate required to attain a target arterial attenuation of approximately 450 HU. This scan used the same infusion rate as the real scan (5 mL/s); however, with half the contrast volume (25 mL), comprising 40% diluted CM, followed by a 25 mL saline chaser at the same injection rate.

**Fig. 1:**
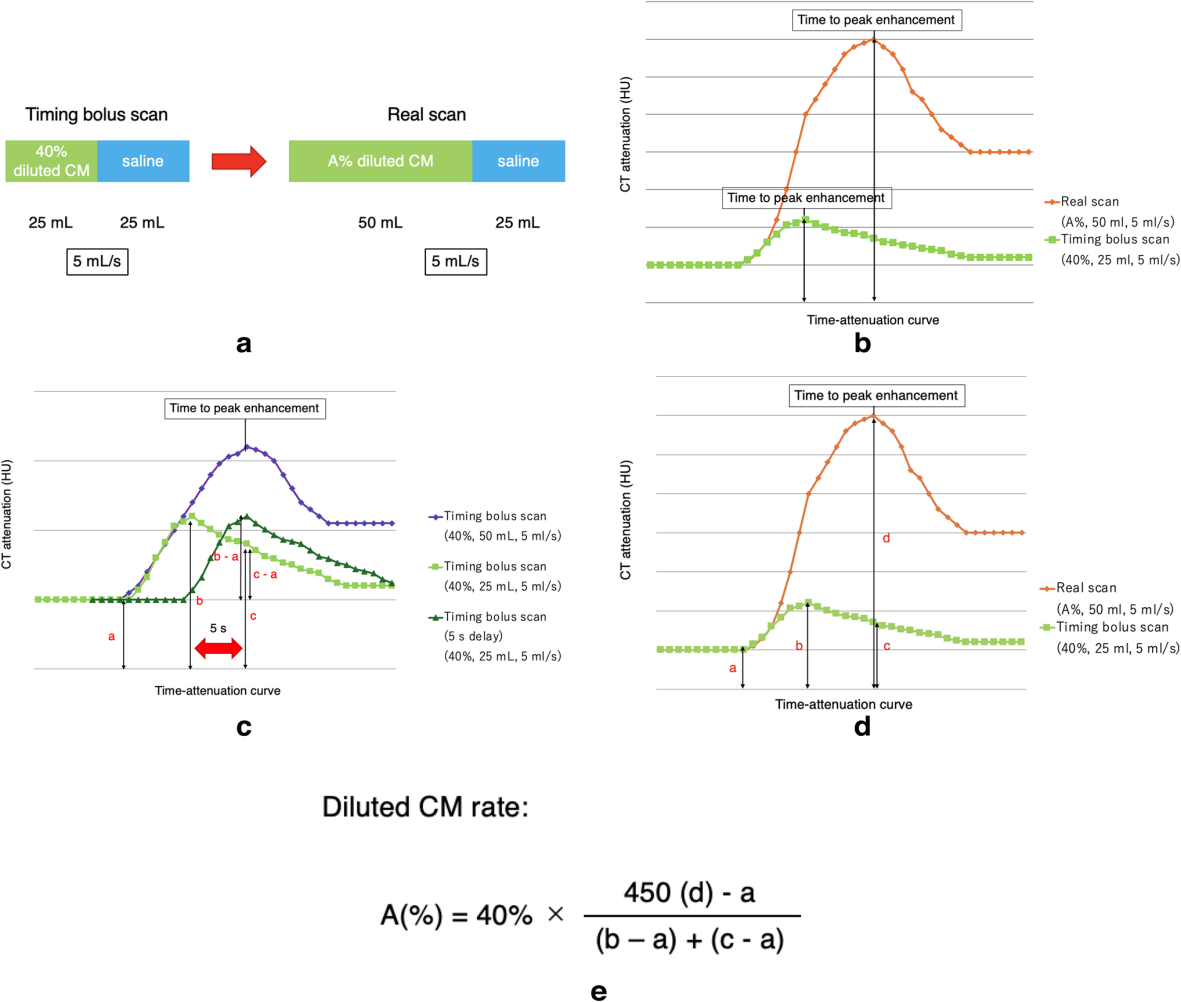
Contrast-injection protocol with diluted CM. **a** Schematic diagram illustrating the contrast-injection process using diluted CM. **b** TAC of the timing bolus scan (lime green) and real scan (orange). **c** Simulation of the TAC with peak enhancement occurring at the same timing as the real scan. In this model: *a* baseline attenuation; *b* peak attenuation, *c* attenuation 5 s after peak. **d** TAC of the timing bolus scan (lime green) and real scan (orange). *a* Baseline attenuation; *b* peak attenuation of timing bolus scan; *c* attenuation 5 s after peak of timing bolus scan; *d* estimated peak attenuation (target attenuation 450 HU); and *e* formula used to determine the optimal CM dilution rate. The optimal scan timing and necessary CM dilution rate (A%) are calculated based on the TAC of the timing bolus scan with 40% CM dilution. *CM* contrast material, *TAC* time attenuation curve, *HU* Hounsfield units

Subsequently, arterial phase time-attenuation curves (TACs) were generated using a series of axial CT images at the level of the ascending aorta in the region of interest (ROI). Owing to varying injection volume and time between the bolus scan and the real scan, the time to peak enhancement does not directly correspond between the two (Fig. 1a and b) [14]. Consequently, determining the appropriate CM dilution ratio requires simulating a TAC in which the peak enhancement occurs at the same timing as in the real scan (Fig. 1c). In this simulation, we assumed that an additional injection of 40% diluted CM (5 mL/s, 25 mL) was administered 5 s after the initial injection. By combining these two injections, the simulated TAC reflected the administration of 50 mL of 40% CM over 10 s. In this model, the change in CT attenuation from the baseline (*a*) at the time of peak enhancement is calculated as the sum of (*b* − *a*) and (*c* − *a*), where:

*a*: Baseline attenuation.

*b*: Peak attenuation.

*c*: Attenuation 5 s after peak.

Therefore, the total increase in CT values above baseline for the simulated 40% 50 mL injection is expressed as (*b* − *a*) + (*c* − *a*). According to a simple linear additive model of mathematical analysis, when the injection rate is identical between the timing bolus and real scan, the contrast volume and resulting enhancement are assumed to be proportional [15]. This relationship can be represented by the following formula:

$$A (\%) : 40\% = (450 - a) : (b - a) + (c - a)$$

Accordingly, the CM dilution ratio (A%) required to attain a target arterial attenuation of 450 HU was calculated using the following formula (Fig. 1e):

$$A (\%) = 40\% \times (450 - a) / \{\left(b - a\right)+ \left(c - a\right)\}$$

A previous study indicated that the optimal timing in a real scan is approximately 3 s before the peak enhancement observed in the TAC [12]. Considering that peak enhancement in this study occurred 5 s earlier in the bolus scan, the optimal scan timing for the real scan was set to 2 s after the TAC peak. This timing was automatically calculated using the formula registered in the CCTA database. The scan duration was approximately 4–8 s. The optimal CM dilution ratio for the real scan was regulated in 5% increments on a dual-injector monitor and implemented clinically promptly. Furthermore, after configuring the optimal CM dilution ratio on the injector, a real scan was conducted using a personalized CM injection protocol (A%, 5 mL/s, 50 mL), followed by a saline chaser (5 mL/s, 25 mL).

### FD protocol

The amount of CM injected (*B* mL) in the actual scan was calculated using the FD protocol. Approximately 22–26 mg iodine/kg/s is recommended for CCTA performed at a tube voltage of 120 kVp [16–18]. CT imaging at a tube voltage of 100 kVp increases iodine CT attenuation by approximately 1.2–1.3 times compared with imaging at a tube voltage of 120 kVp [19–21]. Therefore, in this study, FD = 20 mg iodine/kg/s was used, which is the average value of 24 out of the recommended values of 22–26 for FD at a tube voltage of 120 kVp divided by 1.2.

*B* (mL) = FD (20) × body weight (BW) × s/contrast material concentration (320 or 370 mg iodine/mL).

**Fig. 2:**
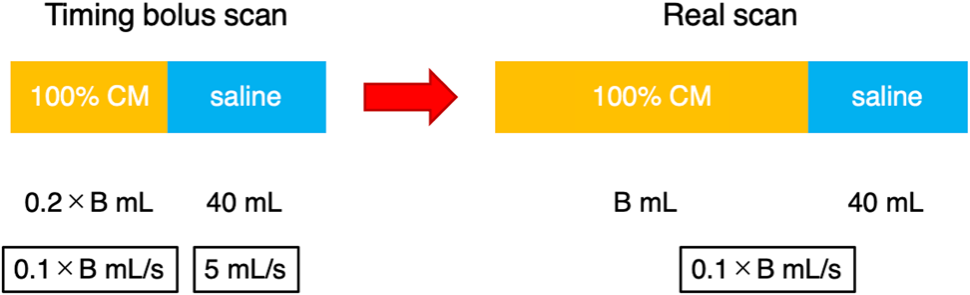
Contrast-injection protocol with FD. Schematic diagram illustrating the contrast-injection process using FD. *FD* fractional dose

First, the timing bolus scan was conducted by injecting 100% CM at one-fifth of the amount of the real scan over 2 s (0.1 × *B* mL/s, 0.2 × *B* mL), followed by a saline chaser (5 mL/s, 40 mL) (Fig. 2). The optimal scan timing for the real scan was set to 9 s after the TAC peak enhancement in the timing bolus scan. The scan duration was approximately 4–8 s. The real scan was conducted by injecting 100% CM of the amount calculated by the FD protocol over 10 s (0.1 × *B* mL/s, *B* mL), followed by a saline chaser (0.1 × *B* mL/s, 40 mL). For clinical use, the injection volume and rate for both the timing bolus and real scans were specified in increments of 1 mL and 0.1 mL/s, respectively.

### Coronary artery attenuation analysis

All CT datasets were analyzed using a dedicated workstation (SYNAPSE VINCENT; FUJIFILM, Tokyo, Japan) by K.O. and K.Y., both cardiovascular imaging experts with 4 and 12 years of experience, respectively. Patients with heart rate ≥ 75 bpm were evaluated at an RR interval of 45%, whereas those with heart rate < 75 bpm were evaluated at an RR interval of 75%.

Using a standard 18-segment model (Society of Cardiovascular Computed Tomography Guidelines), the following segments were identified: the origin of the left main trunk (LM); proximal segment of the left anterior descending coronary artery (pLAD #6); proximal segment of the left circumflex artery (pLCX #11); and proximal, middle, and distal segments of the right coronary artery (pRCA #1, mRCA #2, and dRCA #3, respectively) [22]. The mean aortic attenuation in the ascending aorta was similarly measured using three ROIs at the center of the ascending aorta. All patients underwent measurements of aortic attenuation and six segments of coronary attenuation (LM, pLAD, pLCX, pRCA, mRCA, and dRCA). Coronary segments with severe calcification, diffuse plaque, a diameter ≤ 1.5 mm, or post-stenting were excluded from the analysis. Excluding coronary segments was decided following a consensus between K.O. and K.Y., who have 4 and 12 years of cardiovascular imaging expertise, respectively. Variations in aortic and coronary attenuation were assessed using the standard deviation (SD) × SD metric [12]. In addition, a post hoc power analysis was performed to evaluate the statistical power for detecting differences in both mean attenuation values and attenuation variability between the two protocols.

### Statistical analyses

Normality of data was assessed using the Shapiro–Wilk test. To compare data between the two groups, Student’s t-test was employed for normally distributed data, the Mann–Whitney U test for non-normally distributed data, and Chi-square tests for categorical variables. Differences in the attenuation variability of the aortic and all coronary artery attenuations were assessed using F–tests. Correlations between iodine dose and BW were analyzed using Spearman’s rank correlation coefficient. The Bonferroni correction was applied to adjust for multiple comparisons regarding comparisons of mean attenuation values and variations in the aorta and six coronary segments between the diluted CM and FD protocols. The threshold for statistical significance was set at *P* < 0.0071 (0.05/7). Power analysis was conducted considering the adjusted significance threshold of *P* < 0.0071. A significance threshold of *P* < 0.05 was used for all other comparisons. Statistical analyses were performed using R statistical software (version 4. 4. 1) [23].

## Results

**Fig. 3:**
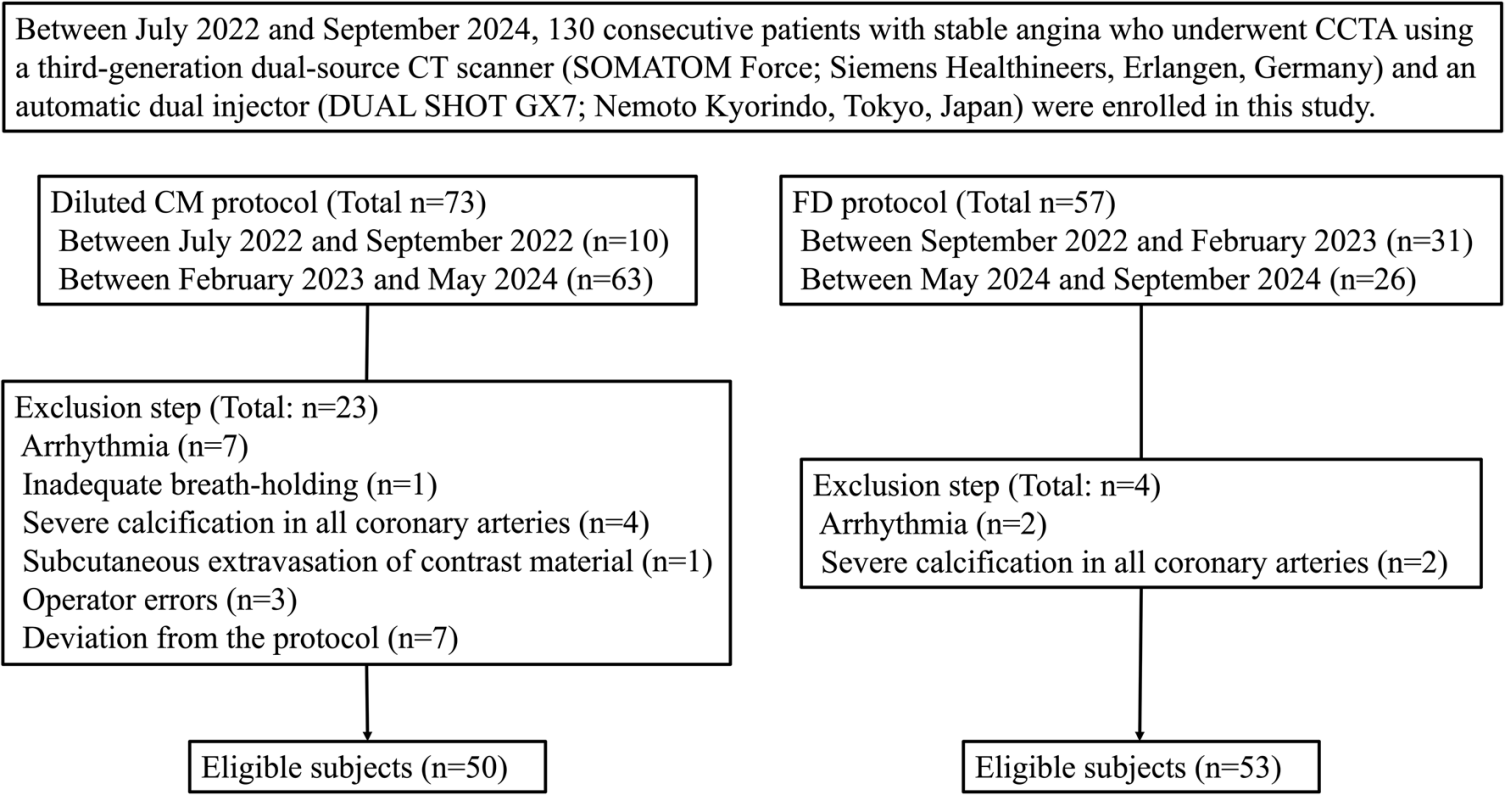
Flow chart for patient selection. *CT* computed tomography, *CM* contrast material, *FD* fractional dose

A total of 130 consecutive patients who underwent CCTA were to be included in this study. Of the 130 patients, 73 underwent CCTA using the diluted CM protocol during two distinct periods: between July 2022 and September 2022 (*n* = 10), and between February 2023 and May 2024 (*n* = 63). The remaining 57 patients underwent CCTA using the FD protocol. This protocol was implemented between September 2022 and February 2023 (*n* = 31) and between May 2024 and September 2024 (*n* = 26). Twenty-seven patients were excluded using predefined criteria. Consequently, 103 patients were included in the final analysis: 50 and 53 in the diluted CM and FD protocol group, respectively (Fig. 3). The baseline characteristics of the patients are summarized in Table 1. The two groups showed no significant differences. The CM injection parameters are listed in Table 2. In the diluted CM and FD protocols, ioversol (320 mg iodine/mL) was used in 37 and 34 patients, respectively. In contrast, iopamidol (370 mg iodine/mL) was administered to 13 and 19 patients, respectively (*P* = 0.30). The median iodine dose per BW in the diluted CM protocol was significantly higher than that in the FD protocol (216 vs. 197 mg iodine/kg) (*P* < 0.001). In the diluted CM protocol, the mean CM dilution rates for ioversol and iopamidol were 71.7 ± 13.3 and 85.1 ± 11.1, respectively. None of the patients required a CM dilution rate exceeding 100%.

**Table 1:** Baseline characteristics

	Diluted CM protocol (*n* = 50)	FD protocol (*n* = 53)	*P* value
Age (years)	72 (65–76)	72 (62–76)	0.96
Male sex (*n*, %)	25 (50%)	29 (55%)	0.70
Body weight (kg)	59.9 ± 12.9	60.7 ± 12.6	0.74
BMI (kg/m^2^)	23.8 ± 3.4	23.6 ± 3.5	0.78
Risk factor (*n*, %)			
Hypertension	28 (56%)	22 (42%)	0.17
Diabetes mellitus	13 (26%)	12 (23%)	0.82
Dyslipidemia	28 (56%)	20 (38%)	0.08
Family history of heart disease	18 (32%)	15 (28%)	0.68
Smoking	25 (50%)	20 (38%)	0.24
Chest pain (*n*, %)	28 (56%)	31 (59%)	0.84

Data are given as the mean ± standard deviation, median (25th–75th percentile), or the number (%) of participants

*CM* contrast material, *FD* fractional dose, *BMI* body mass index

**Table 2:** CM injection parameters during the real scan

	Diluted CM protocol (*n* = 50)	FD protocol (*n* = 53)	*P* value
Injection rate (mL/s)	5	3.5 (3.1–3.8)	< 0.001
Scan delay (s)	21 (19–23)	21 (19–22)	0.53
Scan time (s)	5.3 (4.0–6.0)	5.8 (4.7–6.3)	0.21
Iodine dose (g iodine)	12.4 (10.1–14.6)	11.8 (9.9–13.7)	0.33
Iodine dose per BW (mg iodine/kg)	216 (190–232)	197 (196–199)	< 0.001
Scan heart rate (beats/min)	60.0 (56.0–64.8)	62.0 (59.0–69.0)	0.21

Data are given as median (25th–75th percentile)

*CM* contrast material, *FD* fractional dose, *BW* body weight

### Aortic and coronary attenuation

**Fig. 4:**
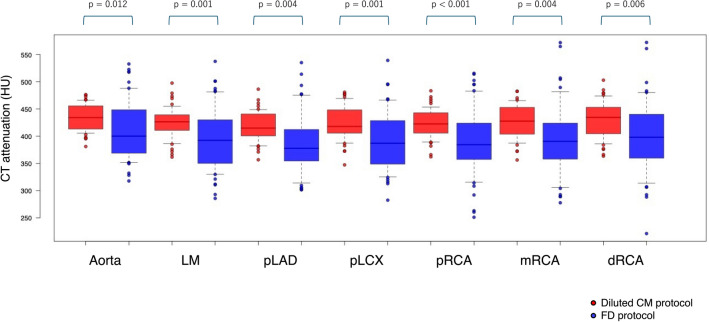
Aortic and coronary attenuations in the diluted CM (red) and FD (blue) protocols. Mean attenuation ± SD are shown for the ascending aorta; origin of the LM; proximal portion of the LAD (pLAD); proximal portion of the LCX (pLCX); and proximal, middle, and distal portions of the RCA (pRCA, mRCA, and dRCA, respectively). The mean attenuations of the aorta and all coronary segments in the diluted CM protocol were significantly higher than those in the FD protocol (*P* < 0.05). Moreover, the variations in the mean attenuation (SD) of the aorta and all coronary artery segments were lower in the diluted CM protocol than in the FD protocol. *CM* contrast material, *FD* fractional dose, *SD* standard deviation, *LM* left main trunk, *LAD* left anterior descending artery, *LCX* left circumflex artery, *RCA* right coronary artery

Twenty-eight coronary segments (pLAD, *n* = 5; pLCX, *n* = 4; pRCA, *n* = 4; mRCA, *n* = 9; dRCA, *n* = 6) in the diluted CM protocol and 28 segments (LM, *n* = 1; pLAD, *n* = 8; pLCX, *n* = 5; mRCA, *n* = 7; dRCA, *n* = 7) in the FD protocol were excluded from analysis because of severe calcification, diffuse plaque, small vessels, or post-stenting. The total number of analyzed segments was 272 and 290 for the diluted CM and FD protocol, respectively. No significant difference was observed in aortic attenuation (434.2 ± 24.2 HU vs. 412.9 ± 54.8 HU; *P* = 0.012); however, the mean coronary attenuation across all coronary segments was significantly higher in the diluted CM protocol group than in the FD protocol group (424.0 ± 26.0 HU vs. 393.0 ± 59.4 HU, *P* < 0.001). Analysis by coronary artery segment, in the diluted CM and FD protocols, yielded the following values: 424.8 ± 28.4 HU and 393.9 ± 58.4 HU in LM, 417.2 ± 26.9 HU and 388.6 ± 57.4 HU in pLAD, 422.1 ± 31.1 HU and 391.1 ± 54.9 HU in pLCX, 421.9 ± 26.2 HU and 390.2 ± 64.3 HU in pRCA, 428.0 ± 32.2 HU and 393.5 ± 69.4 HU in mRCA, and 430.8 ± 33.0 HU and 398.8 ± 68.3 HU in dRCA, respectively (Fig. 4). All coronary attenuations in the diluted CM protocol were significantly higher than those in the FD protocol (*P* < 0.0071). In addition, both variations (SD × SD) in the aortic and all coronary attenuations in the diluted CM protocol were significantly lower than those in the FD protocol (*P* < 0.001), indicating more consistent and uniform enhancement with this protocol. Furthermore, the overall trend remained unchanged after an additional analysis was conducted, including the seven patients who were excluded from the diluted CM protocol group due to protocol deviations (contrast volume > 50 mL in the real scan) (Online resource Fig. 1).

In addition, we conducted a post hoc power analysis using the Bonferroni-adjusted significance level (*P* < 0.0071) for the seven evaluated regions (aorta and six coronary segments) to assess the reliability of these comparisons. The statistical power to detect differences in mean attenuation values was as follows: aorta (0.433), LM (0.753), pLAD (0.632), pLCX (0.744), pRCA (0.664), mRCA (0.588), and dRCA (0.546). In contrast, the power to detect differences in attenuation variability (SD × SD) was high for all segments: aorta (0.998), LM (0.997), pLAD (0.997), pLCX (0.995), pRCA (0.998), mRCA (0.997), and dRCA (0.997).

### Iodine dose and BW in the diluted CM protocol

**Fig. 5:**
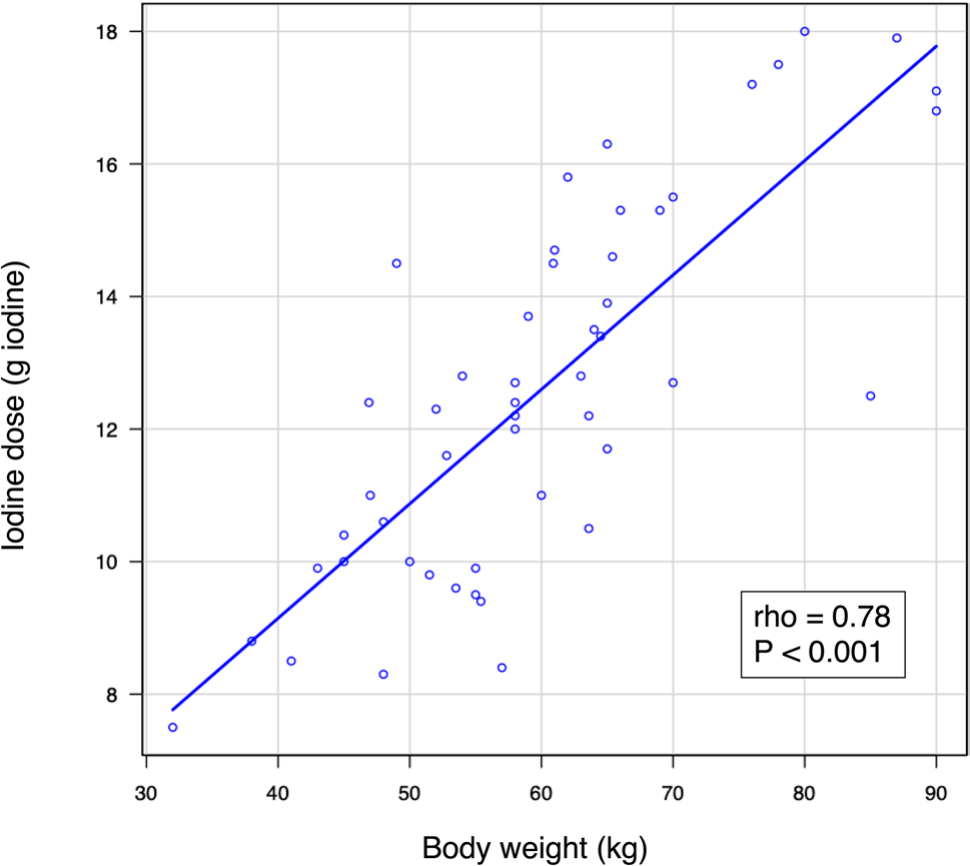
Correlation between iodine dose and BW in the diluted CM protocol. A strong positive correlation was observed between BW and iodine dose (rho = 0.78, *P* < 0.001). *BW* body weight, *CM* contrast material

**Fig. 6:**
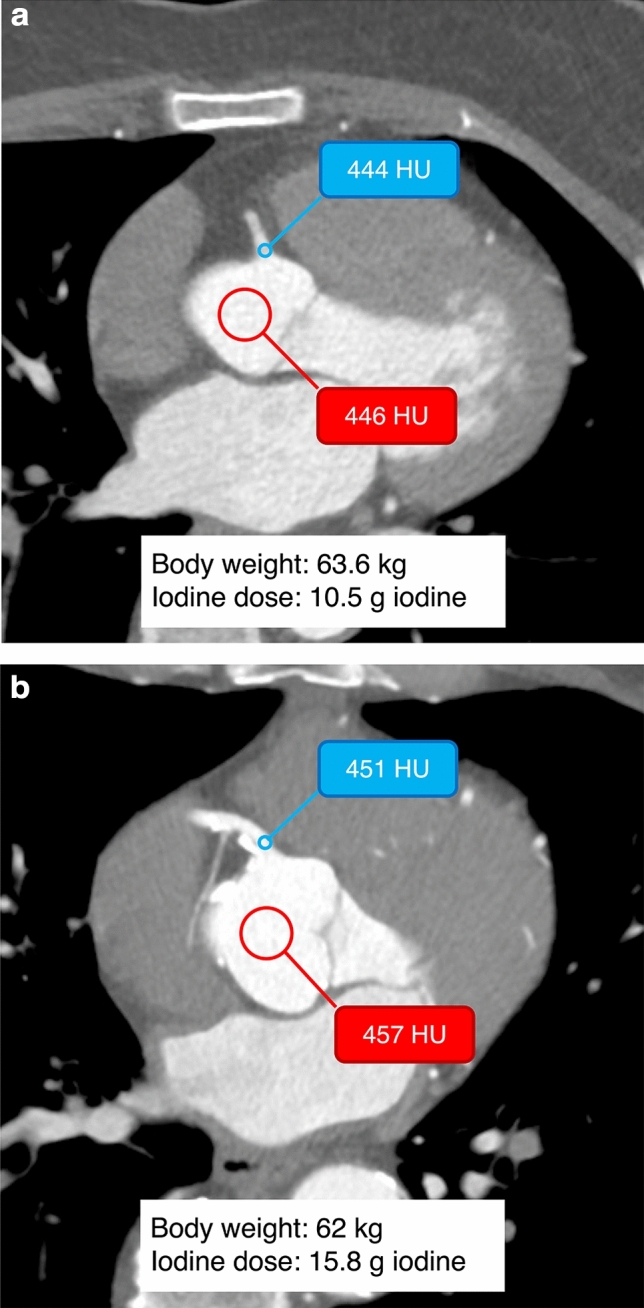
Representative cases of the diluted CM protocol. Axial images of the proximal RCA. **a** A 59-year-old woman (body weight, 63.6 kg; iodine dose, 10.5 g iodine). **b** A 71-year-old man (body weight, 62 kg; iodine dose, 15.8 g iodine). Although the iodine dose per body weight differed between these two patients, similar coronary and aortic attenuations close to the target attenuation were achieved in both cases. *CM* contrast material, *RCA* right coronary artery

Figure 5 illustrates the correlation between iodine dose and body weight (BW) in the diluted CM protocol. A strong positive correlation was observed between BW and iodine dose (rho = 0.78, *P* < 0.001), despite the iodine dose being calculated independently of BW. Despite similar BW, the actual CM dilution ratios and iodine doses in the real scans varied among patients. Figure 6 presents representative cases with comparable BWs but different iodine doses using the diluted CM protocol.

## Discussion

In this study, we demonstrated that implementing a diluted CM protocol considerably enhances the consistency of coronary artery enhancement compared with the conventional FD protocol. Both protocols achieved clinically acceptable mean coronary artery enhancement; however, the diluted CM protocol yielded more consistent aortic and coronary artery enhancement, with notably lower inter-patient variability across all evaluated segments. The statistical power to detect differences in mean attenuation values varied from moderate to low across the coronary segments; however, statistically significant differences were observed (*P* < 0.0071). Nevertheless, the modest power underscored the need for cautious interpretation and further validation in larger studies. In contrast, one of the primary objectives of this study, which was assessing the uniformity of contrast enhancement, consistently yielded robust results. The diluted CM protocol group exhibited significantly lower variability in all coronary segments (*P* < 0.001) using the squared standard deviation (SD × SD) as a metric, with corresponding statistical power exceeding 0.995 across the board. This robust statistical power supports the conclusion that the diluted CM protocol significantly improves the consistency of coronary artery enhancement. These findings are consistent with those of a previous study that demonstrated improved uniformity of coronary enhancement using the diluted CM technique at 120 kVp [12]. Our study extends this evidence to a 100 kVp setting—currently recommended for patients with a BMI of < 30—demonstrating that the benefits of reduced variability remain valid under lower tube voltage conditions [13]. The diluted CM protocol may be beneficial for maintaining the consistency of the coronary artery enhancement regardless of the tube voltage. One possible reason for this reduced variability is the individualized adjustment of the contrast material dilution rate based on the patient's TAC. As shown in Fig. 6, despite similar body weights, the administered iodine doses varied between patients, potentially reflecting differences in cardiac function, body surface area, or BMI [12, 14, 24–26]. Such patient-specific optimization may contribute to the improved consistency in coronary artery enhancement observed with the diluted CM protocol. The ability to preserve image quality while reducing radiation dose enhances the overall safety and utility of CCTA in routine clinical practice.

Moreover, consistent coronary artery enhancement is particularly beneficial for evaluating plaques and FAI. Previous studies have shown that coronary artery enhancement influences the assessment and quantification of plaque components [19, 27, 28] and that consistent enhancement is essential for accurate plaque characterization. Furthermore, FAI values, which are increasingly used as noninvasive markers of perivascular inflammation [9], vary depending on luminal enhancement [29, 30]. By providing consistent coronary artery enhancement, the diluted CM protocol may ensure that FAI measurements are more reproducible and reliable, thereby strengthening their role in predicting future cardiac events and guiding preventive therapies. Moreover, consistent coronary artery enhancement enhances the reliability of both intra- and inter-patient comparisons. This is particularly important in longitudinal clinical practice and studies, such as follow-up evaluations of plaque progression or regression, where minimizing variability in imaging conditions is essential for interpreting subtle changes [31, 32]. It also facilitates standardized inter-patient assessments in research and clinical trials by reducing contrast-related heterogeneity.

A previous study compared the diluted CM and BW-adjusted protocols using contrast material diluted to the same concentration, thereby standardizing the conditions for the timing of the bolus scan [12]. In contrast, this study utilized a 100% contrast material in the timing bolus scan, based on a commonly implemented FD protocol. This difference in the comparator protocol allowed a comparison that closely reflected routine clinical practice and may have enhanced the clinical relevance of the findings. An additional advantage of the diluted CM protocol is the improved workflow efficiency. A lower total volume of saline (timing bolus scan: 40 mL, real scan: 25–75 mL) was used compared to the conventional diluted CM protocol (timing bolus scan: 80 mL, real scan: 40–90 mL), which reduced the need to switch saline syringes between phases. This may reduce the preparation time and the potential for technical errors, which is particularly beneficial in high-throughput clinical settings.

## Limitations

This study has some limitations. First, the relatively small sample size and single-center design may limit the findings’ generalizability. Different institutions with varying patient populations, imaging protocols, patient characteristics, and equipment capabilities might not replicate these results. Larger and more diverse multicenter studies help validate and extend these findings across broader clinical settings. Second, the diluted CM protocol tended to require a slightly higher iodine dose per body weight compared to the FD protocol. This difference did not result in any adverse events in our cohort, likely because most patients undergoing CCTA had preserved renal function. However, in patients with pre-existing renal dysfunction or those requiring repeated contrast-enhanced imaging, the FD protocol may be a more suitable method in such cases. Third, the diluted CM protocol requires a fixed injection rate of 5 mL/s, which necessitates the use of at least a 20-gauge intravenous catheter. While the use of a 20-gauge intravenous catheter is typically recommended, secure placement may not be feasible in patients with fragile veins, such as older adults or those with prior chemotherapy or vascular access issues. Lowering the injection rate to accommodate smaller-gauge catheters could impair contrast enhancement, potentially offsetting the benefits of the diluted CM protocol. Moreover, the fixed injection rate of 5 mL/s may not be well tolerated in patients with fragile veins and carries a risk of subcutaneous extravasation. Notably, the difference was not statistically significant; however, one case of contrast agent extravasation occurred in the diluted CM protocol group. In contrast, no such cases were observed in the FD protocol group. Fourth, implementing the diluted CM protocol requires access to automated injector systems that can adjust dilution ratios and timing in real-time. Facilities lacking such advanced equipment may face practical challenges in adopting this approach, limiting its widespread applicability. Fifth, the volume of saline chaser differed between the diluted CM and FD protocols (25 mL vs. 40 mL in the diluted CM and FD protocol group, respectively). The difference in saline volume may have influenced the peak enhancement and the shape of the TAC, potentially affecting the observed contrast dynamics. Furthermore, the amount of saline chaser used in the diluted CM protocol was slightly lower than the 30–50 mL recommended by the Society of Cardiovascular Computed Tomography guideline [13]. This volume met the minimum requirement for achieving a basic flushing effect, as reported by Yamaguchi et al. [33]; however, it may have been less effective in maximizing contrast enhancement and in reducing imaging artifacts caused by residual high-concentration contrast material in central veins, particularly beam-hardening artifacts around the superior vena cava and right atrium. Notably, the diluted CM protocol achieved adequate coronary contrast enhancement despite the reduced saline volume. Finally, this study did not directly evaluate the impact of reduced enhancement variability of coronary plaque characteristics or FAI assessments. A definite comparison would ideally involve applying both the diluted CM and FD protocols to the same patients under identical conditions. However, performing two separate CCTAs on the same individual is often impractical. Consequently, although the reduced variability observed in the diluted CM protocol suggests potential benefits for plaque and FAI evaluation, this remains speculative and warrants further dedicated study.

## Conclusions

The diluted CM protocol demonstrated substantially more consistent coronary enhancements compared with the FD protocol during coronary CT at a tube voltage of 100 kVp. The diluted CM protocol effectively reduced the variability in contrast enhancement, which may enhance the reliability of coronary artery stenosis assessment, potentially enhancing diagnostic accuracy and clinical decision-making.

## Supplementary Information

Below is the link to the electronic supplementary material.

Supplementary file1 (TIFF 191414 KB)

Supplementary file2 (DOCX 17 KB)
