# Supplementary material for: Coronary computed tomography angiography using the diluted contrast material protocol: a technique for achieving uniform coronary artery enhancement
Source: Jpn J Radiol. 2025 Jul 30;43(12):1971–80. doi: 10.1007/s11604-025-01845-y (PMC12647354; doi:10.1007/s11604-025-01845-y)
Supplement: Supplementary file 2 — Supplementary file2 (DOCX 17 KB) [file 11604_2025_1845_MOESM2_ESM.docx]

**Article title: Coronary Computed Tomography Angiography using the Diluted Contrast Material Protocol: A Technique for Achieving Uniform Coronary Artery Enhancement**

**Journal name**: Japanese Journal of Radiology

**Author names**: Kentaro Ohara, MD^1^; Kazuki Yoshida, MD, PhD^1^; Hikaru Nishiyama, MS^1^; Yuki Tanabe, MD, PhD^1^; Yusuke Kobayashi, MD^1^; Naoto Kawaguchi, MD, PhD^1^; Megumi Matsuda, MD, PhD^1^; Kaito Okamoto, MD^1^; Shiori Utsunomiya, MD^1^; Teruhito Kido, MD, PhD^1^

**Affiliation**: ^1^Department of Radiology, Ehime University Graduate School of Medicine, Toon, Ehime, Japan

**Email addresses**: kntr753@gmail.com

**Supplementary Table 1. Baseline characteristics**

|  | Diluted CM protocol  (n = 29) | FD protocol  (n = 42) | *P*-value |
| --- | --- | --- | --- |
| LVEF (%) | 63.0 (57.6–66.7) | 61.6 (59.5–67.0) | 0.79 |
| LVEDV (mL) | 67.3 (57.0–82.7) | 69.6 (50.6–84.6) | 0.79 |
| LVESV (mL) | 27.1 (19.7–31.5) | 26.6 (18.6–32.4) | 0.64 |

Data are given as the median (25th-75th percentile)

Twenty-one patients from the diluted CM protocol and 11 patients from the FD protocol were excluded from the analysis due to missing echocardiographic data within 6 months before or after CCTA

*CM,* contrast material; *FD,* fractional dose; *LVEF,* left ventricular ejection fraction; *LVEDV*, left ventricular end-diastolic volume; *LVESV*, left ventricular end-systolic volume, *CCTA*, coronary computed tomography angiography

**Supplementary Fig.1**: Supplementary analysis of aortic and coronary attenuations in the diluted CM (red) and FD (blue) protocols

Mean attenuation ± SD are shown for the ascending aorta; origin of the LM; proximal portion of the LAD (pLAD); proximal portion of the LCX (pLCX); and proximal, middle, and distal portions of the RCA (pRCA, mRCA, and dRCA, respectively). The mean attenuations of the aorta and all coronary segments in the diluted CM protocol were significantly higher than those in the FD protocol (P < 0.05). Moreover, the variations in the mean attenuation (SD) of the aorta and all coronary artery segments were lower in the diluted CM protocol than in the FD protocol.

**Abbreviations:** *CM,* contrast material; *FD,* fractional dose; *SD,* standard deviation; *LM,* left main trunk; *LAD,* left anterior descending artery; *LCX,* left circumflex artery; *RCA,* right coronary artery
